# Successful Management of Aluminum Phosphide (Celphos) Poisoning With Aspiration Pneumonia: A Case Report

**DOI:** 10.1002/ccr3.70228

**Published:** 2025-02-20

**Authors:** Rakshya Arun Kandel, Rukma Raj Kafle, Sabin Chaulagain, Chandan Shah, Alok Atreya

**Affiliations:** ^1^ Department of Emergency Medicine Scheer Memorial Adventist Hospital Banepa Nepal; ^2^ Department of Internal Medicine Scheer Memorial Adventist Hospital Banepa Nepal; ^3^ Vayodhya Hospital Kathmandu Nepal; ^4^ Department of Forensic Medicine Lumbini Medical College Palpa Nepal

**Keywords:** aluminum phosphide, antidotes, coconut oil, gastric lavage, metabolic acidosis, Nepal, potassium permanganate, suicide

## Abstract

Aluminum phosphide (AlP) poisoning typically has a poor prognosis, but this case report from Nepal demonstrated that prompt hospitalization, aggressive supportive care, including gastric lavage with coconut oil, and careful management of metabolic acidosis can lead to successful patient recovery.

## 1 Introduction

Pesticide poisoning accounts for approximately 300,000 fatalities annually worldwide, with organophosphates and phosphides being predominant agents [1]. Aluminum phosphide (AlP) is particularly concerning due to its accessibility, lack of a specific antidote, and frequent use in suicides [1]. AlP is widely used as an effective fumigant for preserving grains, with one of the most lethal toxicity profiles among pesticides, with mortality rates exceeding 65% in the case of ingestion [2]. It is marketed under names such as Synfume, Phostek, Phostoxin, Phosfume, Celphos, Alphos, and Quickphos [3, 4]. It comes as a dark gray tablet in the amount of 3 g [4]. Due to its easy availability and sales in the open market, it is one of the most frequently used agents for self‐poisoning in several developing countries, including Nepal [3, 4]. Demography on poisoning patterns depicts a young population from rural areas being the more common victims [3, 4].

The review by Sharma et al. (2012) estimated that the annual import of pesticides in Nepal was approximately 211 tons, among them one of the highly hazardous pesticides (WHO class Ib) that had been banned by some countries is AlP, which is still legally sold [5]. The lack of knowledge among farmers using AlP without proper training results in a large number of deaths due to respiratory failure [5]. The overall prognosis of AlP poisoning is grave; clinical evidence on successful management and improved survival outcomes after treating AlP poisoning cases has been scarce, with only a handful of literatures available to date [1, 6, 7].

This case report describes the successful treatment and recovery of a 47‐year‐old male patient who presented with AlP ingestion and developed aspiration pneumonia, demonstrating that with prompt hospitalization and appropriate supportive interventions, AlP poisoning can be effectively managed even in a developing country setting. The findings from this case report aim to raise awareness about the possibility of successful treatment outcomes for AlP poisoning and the importance of early intervention and comprehensive care.

## 2 Case History

A 47‐year‐old male presented to the Emergency Department (ED) after deliberately ingesting one fresh aluminum phosphide tablet immediately after tea, 4 h prior to arrival. He experienced vomiting and mild abdominal pain localized around the periumbilical region.

## 3 Methods

### 3.1 Initial Assessment and Decontamination

At the ED, he was semiconscious, with a Glasgow Coma Scale (GCS) of 13/15, responding to painful stimuli and a blood pressure of 90/60 mmHg (Table 1). His oxygen saturation in room air was 92%. Immediate decontamination and gastric lavage with coconut oil were performed, and IV crystalloids were started.

**TABLE 1:** Vitals of the patient at the time of presentation to discharge.

Variables	At ED	Day 1	Day 2	Day 3	Day 4	Day 7	Reference range
Blood pressure (BP) (mmHg)							
Systolic	90	80–100	100–120	110–140	110–130	120–130	100–120
Diastolic	60	50–60	60–70	60–80	70–90	70–80	80–100
Heart Rate (beats/min)	114	82–100	72–86	60–72	66–68	70–78	60–100
Oxygen saturation (%) in room air	90–92	85–88	89–94	94–98	94–98	96–98	92–96
Temperature (°F)	97.8	99.8	99.6	98.2	98.4	98.2	97–99
Capillary blood glucose (mg/dL)	178	160	122	110	132	94	120–140

### 3.2 Investigation

The results of routine blood tests were within normal limits except for random blood sugar, which was slightly elevated (174 mg/dL). An electrocardiogram (ECG) revealed sinus tachycardia without ST‐T changes. Arterial blood gas (ABG) analysis revealed early metabolic acidosis with pH 7.31, PCO_2_ 17.4 mmHg, and HCO_3_ 19.6 mEq/L.

### 3.3 Physical Examination

The patient had a peculiar odor on breath. Chest auscultation revealed bilateral scattered rhonchi. Cardiovascular examination showed tachycardia with normal heart sounds. Neurological examination demonstrated mild confusion with a focal deficit.

### 3.4 Monitoring and Treatment

The patient was admitted to the intensive care unit (ICU) for further monitoring and treatment, including IV fluids. In the ICU, the BP fluctuated on the lower side, which was corrected by IV fluids. Six hours after admission to the ICU, ABG analysis still revealed metabolic acidosis. The ABG was repeated every 24 h until acidosis had normalized.

**FIGURE 1:**
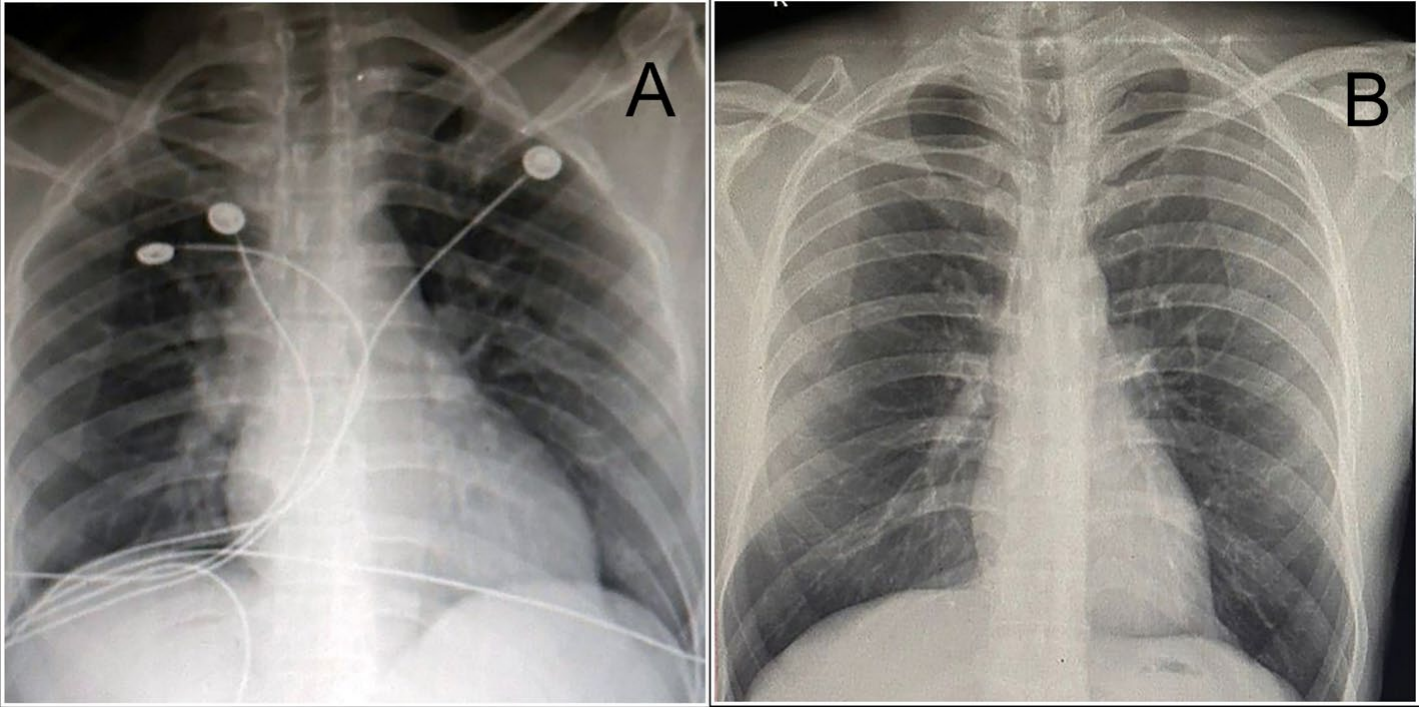
X‐ray of the chest showing (A) consolidation in the right lower zone and (B) improvement after treatment.

On day 1 after admission, the patient developed shortness of breath, and his oxygen saturation decreased. He was immediately placed on a nasal cannula at 2 L/min of oxygen support. He also had a persistent low‐grade fever with a maximum recorded temperature of 99.8°F. An immediate chest X‐ray revealed right lower zone consolidation (Figure 1A), for which antibiotics and steroids were added. Ceftriaxone (2 g) was injected every 24 h, and clindamycin (600 mg) was injected every 8 h via the IV route for 7 days. Two hundred milligrams of intravenous hydrocortisone was given as a static dose, and then 100 mg of hydrocortisone was given every 8 h for 3 days. He was weaned from oxygen support after 24 h.

N‐acetylcysteine (NAC) was administered at a loading dose of 150 mg/kg over 1 h, followed by 50 mg/kg over 4 h, and then 100 mg/kg over 16 h. Magnesium sulfate was supplemented (3 g bolus with maintenance dose of 6 g 12 hourly) to maintain normal levels due to its potential antioxidant properties. Continuous cardiac monitoring showed occasional premature ventricular contractions that resolved spontaneously. Daily liver and renal function tests remained stable throughout the hospital stay.

## 4 Results

### 4.1 Outcome

On day 2 of admission, the NG tube was removed, and peripheral total parenteral nutrition was started initially at 30 cc/h and maintained at 50 cc/h over 24 h. By day 4, the patient tolerated oral feeding and was moved to the general ward, and a psychiatric consultation was conducted. After 1 week, repeat investigations were normal, and a repeat chest X‐ray revealed resolved consolidation (Figure 1B). The patient was discharged with advice to follow‐up instructions, including mental health assessments.

### 4.2 Follow‐Up

Despite recommendations for follow‐up care and mental health assessment, the patient did not return for subsequent appointments. This case was reported to local law enforcement authorities as per medicolegal requirements.

## 5 Discussion

Aluminum phosphide (AlP) poisoning represents one of the most challenging toxicological emergencies with high mortality rates [2]. This case illustrates that early identification, prompt intervention, and comprehensive supportive care can lead to successful outcomes even in severe cases. Understanding the mechanism of action and implementing appropriate treatment strategies were crucial to the survival of the patient.

### 5.1 Mechanism of Action

The grayish‐white 3 g tablet usually contains 44% aluminum carbonate and 56% aluminum phosphide and has the capacity to release 1 g of phosphine gas (PH_3_) [4].

The toxicity of AlP primarily results from the release of PH_3_ when it comes into contact with moisture or hydrochloric acid in the stomach [1]. The exact mechanism of toxicity is complex and multifaceted. Current evidence suggests that phosphine primarily: [1, 8, 9]

- Inhibits cytochrome oxidase, disrupting mitochondrial oxidative phosphorylation.
- Generates reactive oxygen species, leading to oxidative stress
- Causes lipid peroxidation and protein denaturation of cell membranes.
- Reduces glutathione levels, compromising cellular antioxidant defenses.

These mechanisms result in widespread cellular damage, particularly in organs sensitive to low oxygen, such as the heart, lungs, liver, and kidneys. GI symptoms typically develop first after exposure [4]. Symptoms may include nausea, vomiting, abdominal pain, and diarrhea [4]. This was evident in our patient as metabolic acidosis, severe vomiting, and transient hypotension, consistent with the documented toxic effects of AlP [1, 8]. The patient also developed pneumonia, probably due to aspirated vomitus, for which proper medications were started.

When AlP poisoning occurs, metabolic acidosis poses a serious threat to life [4]. This is most likely the result of low tissue perfusion and lactic acid buildup caused by oxidative phosphorylation inhibition. Another prognostic marker for AlP poisoning is the degree of metabolic acidosis [3, 4]. The survival rate in cases of AlP poisoning is significantly affected by complete acidosis treatment [3, 4].

### 5.2 Treatment Approach and Rationale

With a positive history of ingesting AlP tablets, symptoms suggestive of AlP intake, and a garlic‐like odor at presentation, the diagnosis is primarily clinical. There is no known specific antidote for AlP to date. Until phosphine is eliminated from the body, management principles seek to maintain life with proper resuscitation techniques.

The management principles in this case focused on four key therapeutic approaches:

1. Early decontamination
  – Coconut oil was used for prompt gastric lavage because current evidence indicates that coconut oil may reduce phosphine gas absorption [6, 8].
  – The intervention was especially beneficial since the patient had arrived less than 4 h after ingestion.
2. Management of metabolic acidosis
  – Serial ABG monitoring indicated early detection and management of metabolic acidosis [8, 9].
  – Fluid resuscitation is one of the crucial factors that keep all organs perfused and control acidosiss [6, 8, 9].
  – This was essential considering that metabolic acidosis is one of the determinants of mortality in AlP poisoning [8, 9].
3. Hemodynamic Support
  – Early recognition and management of hypotension are achieved with IV crystalloids [8, 9].
  – Continuous monitoring in the ICU setting allowed for prompt intervention when blood pressure fluctuated [6, 7].
  – This was particularly important as cardiovascular collapse is a common cause of death in AlP poisoning [8, 9].
4. Management of complications
  – Prompt recognition and treatment of aspiration pneumonia with appropriate antibiotics (Ceftriaxone and Clindamycin) and steroids.
  – Supplemental oxygen support when needed.
  – Sequential progression from parenteral to oral nutrition as the patient improved.

The positive outcome in this case can be attributed to several factors:

- Early presentation to the hospital (within 4 h of ingestion)
- Immediate initiation of appropriate decontamination measures
- Comprehensive supportive care in an ICU setting
- Systematic monitoring and management of complications
- Holistic approach including mental health assessment

This case adds to the limited literature demonstrating successful outcomes in AlP poisoning and highlights the importance of prompt, systematic, and comprehensive care. While AlP poisoning continues to have a high mortality rate, with appropriate interventions, favorable outcomes are achievable, as demonstrated by our experience even in resource‐limited settings.

### 5.3 Limitations and Future Directions

The present case report has inherent limitations due to its design. Being a single case study, the results are not generalizable. Insufficient data regarding long‐term follow‐up is also a limitation. Patients do not turn up for follow‐up visits due to the social stigma of suicide attempts in Nepal. The requirement for police reporting in such medicolegal cases may have deterred the patient from seeking follow‐up care.

Future research directions should focus on:

- Development and validation of standardized treatment protocols for AlP poisoning, especially in resource‐limited settings
- Evaluation of antioxidant therapy including glutathione, vitamins C and E, carotenoids, and melatonin in improving survival outcomes [10]
- Assessment of long‐term physical and psychological consequences in survivors
- Investigation of potential antidotes through systematic research
- Study of social interventions to improve follow‐up compliance and reduce stigma
- Analysis of the impact of mandatory legal reporting on treatment

## 6 Conclusion

Since AlP poisoning has a poor prognosis, patients who suffer from it rarely recover, particularly in underdeveloped nations such as Nepal. Recovery is achievable with prompt hospitalization and aggressive supportive care. We were able to save the patient in this uncommon instance where he had nonspecific symptoms in addition to hypotension and metabolic acidosis following AlP consumption. In our case, the patient made it through prompt presentation and early intensive supportive care. It is imperative that authorities show a strong interest in not allowing vendors and shopkeepers to sell AlP tablets without proper verification and confirmation.

## Author Contributions

**Rakshya Arun Kandel:** data curation, validation, writing – original draft. **Rukma Raj Kafle:** data curation, writing – review and editing. **Sabin Chaulagain:** data curation, writing – review and editing. **Chandan Shah:** writing – original draft, writing – review and editing. **Alok Atreya:** conceptualization, supervision, writing – review and editing.

## Consent

Written informed consent was obtained from the patient to publish this report in accordance with the journal's patient consent policy.

## Conflicts of Interest

The authors declare no conflicts of interest.

## Data Availability

All data underlying the results are available as part of the article, and no additional source data are needed.
